# Supplementary material for: Are carbon emissions trading and green financial instruments synergistic? -Comprehensive quantitative research based on content analysis
Source: PLoS One. 2024 Mar 7;19(3):e0298601. doi: 10.1371/journal.pone.0298601 (PMC10919601; doi:10.1371/journal.pone.0298601)
Supplement: S1 Data — (ZIP) [file pone.0298601.s001.zip › Data/地级市Z_F工作报告.zip!/║■─╧/┬a╡╫╩╨/└┤╫╘╣1⁄2╓┌║┼í╛┬φ┐╦╩2╛▌═°í┐_2017.docx]

各位代表：

　　现在，我代表市人民政府，向大会作工作报告，请予审查，并请各位政协委员和列席人员提出意见。

　　一、过去四年和2016年工作回顾

　　过去的四年，是宏观经济形势极其复杂、我市发展极不平凡的四年，是我市经受重大挑战、并取得丰硕成果的四年。在市委的坚强领导下，全市上下深入贯彻党的十八大和十八届三中、四中、五中、六中全会精神，认真落实省委、省政府的决策部署，主动适应发展新常态，深入践行发展新理念，大力实施融城发展、娄商返娄、对接大长沙战略，全面完成六个“四年行动计划”，部署推进七个“三年行动计划”，努力开创了全市经济社会发展的新局面，奋力谱写了“加速转型、奋力赶超”的新篇章。

　　综合实力明显提升。地区生产总值达到1406亿元(预计数，下同)，年均增长8.6%；固定资产投资达到1190亿元，年均增长21.4%；一般公共预算收入、地方收入分别达到104.58亿元、68.13亿元，年均分别增长4.2%、7.1%；社会消费品零售总额达到485亿元，年均增长12.2%。六大高耗能行业增加值占比下降12.6%，高技术、高加工度产业年均分别增长12.5%、16.8%，特色农业、商贸物流、文化旅游、现代金融快速发展，三次产业结构调整为14.7:48.4:36.9。娄底经开区晋升为国家级园区，我市园区综合排名从全省第10跃升到第5，新化县、双峰县、涟源市跻身全省特色县域经济重点县。

　　城乡面貌明显变化。沪昆高铁、娄邵铁路(扩能改造)建成通车，高速公路建成5条、在建2条，总里程达到434公里，新建(改造)国省干线公路395公里、乡村公路2780公里，娄底大道娄涟段基本贯通，我市的空间经济地理格局正在发生根本变化。城乡统筹取得积极进展，全市城镇化率达到45.27%，交通、水利、能源、信息“四张网”建设快速推进，全市24个村被评为全省新农村建设示范村和美丽乡村示范村，冷水江市在全省率先整市推进新农村建设。全面推进城乡环境整建和“九大环保工程”，圆满完成湘江保护和治理第一个三年行动计划，在全国率先开展领导干部自然资源资产离任审计，与湘潭市签署《共同保护水府庙行动宣言》，新化县纳入全国重点生态功能区。全市森林覆盖率达到50.21%，中心城区空气质量优良率达到87.7%，全市9个监测断面的地表水质均达到Ⅲ类以上，7个县级集中式饮用水源全部达标，农村环境质量排名由全省第7位上升到第3位。圆满承办第十二届省运会和第九届省残运会，市本级成功创建国家园林城市和国家卫生城市，获得创建全国文明城市提名。

　　发展活力明显增强。扎实推进“三去一降一补”，供给侧结构性改革初见成效。政府机构、合村并乡等改革全面完成。司法体制、营改增、放管服、不动产统一登记、农村土地承包经营权确权登记和抵押贷款试点、全国编制自然资源资产负债表试点等改革全面推进。投融资体制改革取得突破，成功组建娄底农商行，引进5家商业银行，4家企业在新三板挂牌。在全省率先出台深化国企改革实施意见。先后成立我市首家省级重点实验室、首家院士工作站、5家工程技术研究中心，建成2个省级示范众创空间、8个创业孵化基地。加快铁路口岸建设，引进海关、商检机构，设立外经外贸综合服务平台，“湘博会”成为省级招商平台，成功举办第一届湖南省汽车配套产业对接会。四年实际利用内资1648亿元、到位外资13.7亿美元，年均分别增长23.6%、14.0%。

　　民生福祉明显改善。民生支出占财政支出的比重达到72%，比2012年提高2.54个百分点。城乡居民人均可支配收入分别达到23606元、9529元，年均增长8.5%、11.0%。全面小康总体实现程度达到87.5%，冷水江市成为全省首批全面小康达标县市。四年新增城镇就业15.9万人、农村劳动力转移就业22.2万人，城镇登记失业率控制在4.19%以下；建设保障性住房10.78万套，改造农村危房3.42万户；建成农村幸福院435个，改扩建敬老院37所；改扩建学校446所，建成合格学校662所。城乡居民基本医疗制度完成整合，大病保险全面实施。公立医院改革全面铺开，分级诊疗试点进展顺利。市级“四馆一中心”建成使用。“菜篮子”工程成效明显。积极创建“全国社会管理综合治理优秀市”“基层组织先进市”，“平安娄底”建设步伐加快，安全生产工作进入全省先进行列。

　　政府建设明显加强。法治政府建设走在全省前列，发布了全省首个政府《行政复议行政应诉》白皮书，全面推行“三张清单”制度，建立了政府法律顾问团队。主动接受市人大法律监督和工作监督，自觉接受市政协民主监督、职能机关专门监督和社会各界舆论监督。四年办结人大代表议案建议816件、政协委员提案637件。主动回应网民诉求，红网留言办复率和满意度逐年提高。扎实开展党的群众路线教育实践活动、“三严三实”专题教育、“两学一做”学习教育和责任心教育，严格落实中央八项规定、省委九项规定，出台提高政府效能“六条规定”和“禁赌限牌”规定，干部的“四种意识”、精神状态、工作作风有了新转变，履职尽责、攻坚克难的能力有了新提高。

　　国防动员和后备力量建设创新加强，军民融合深度发展，全民国防教育普及深化，成功创建“全省双拥模范城”，驻娄解放军、武警、消防、公安和民兵预备役遂行任务能力持续提升，参建参治作用不断显现。统计、审计、政务服务、电子政务、住房公积金、人防、地震、气象等部门，服务发展的能力不断加强。地方志、档案、保密、外事侨务、工商联、民族宗教、对台、贸促、工会、共青团、妇女儿童、老龄、红十字会、关工委、科协、计生协、文联、慈善、残联等各项工作均取得新的进步。

　　2016年，是本届政府的收官之年。一年来，我们坚持加速转型、奋力赶超，以供给侧结构性改革为主线，以提高质量效益为中心，着力谋长远、打基础、抓关键、惠民生，实现了“十三五”的良好开局。主要抓了以下五个方面的工作：

　　一是以钉钉子的精神抓好“十三五”规划的落地落细落实。坚持一张蓝图干到底，部署推进七个“三年行动计划”，下定决心用三年时间把五年的事情干完，开启了我市发展的“加速度”。预计地区生产总值增长8.0%，固定资产投资增长14.1%，社会消费品零售总额增长11.8%，一般公共预算收入、地方收入分别增长9.66%、13.69%，城乡居民收入分别增长8.1%、10.1%，主要经济指标增速在全省排位明显前移。

　　二是狠抓项目建设扩投资、稳增长、增后劲。大力开展“项目建设大比拼”活动，696个重大项目完成投资720亿元，超年度计划15%，新开工、竣工项目分别增长12%、16%。在全省率先出台PPP项目商务条件、工作流程和工作标准，设立专项财政引导基金和城市建设投资基金50亿元，开发PPP项目53个，总投资超过1000亿元，引进了一批“国字号”战略投资者，其中6个已开工或即将开工建设，总投资超过100亿元。

　　三是创新思路出实招、解难题、补短板。出台工业帮扶十五条，设立工业产业、中小企业“助保贷”专项铺底、工业转型发展等引导基金，帮助实体企业渡难关。新增“四上”达标入统企业270家。新增金融机构7家，与4家银行签订战略合作框架协议，新增授信额度500亿元。

　　成功组建国有资本运营、文化旅游发展、交通建设等政府性融资平台，争取国家专项建设基金近80亿元。全面开展房地产业“解难题、促发展、保稳定”专项行动，全市房地产销售面积达235万平方米，商品房库存去化周期控制在16个月以内。

　　四是着眼聚人气、提品质、创一流，加快谋划城镇规划、建设和管理。行政区划调整取得实质性进展，涟源市、双峰县部分镇村并入中心城区的方案，进入省级审批阶段。我市作为除长株潭外的唯一市州，全域纳入了全省新型城镇化战略布局。编制完成了《智慧娄底建设五年行动计划》，中心城区总投资达46亿元的2个PPP项目开工建设，积极推进中心城区废弃铁路综合开发。长沙-娄底-新化低空旅游观光航线成功试飞。高速公路沿线8个特色小镇建设试点全面启动。

　　五是多措并举推动精准扶贫、精准脱贫，着力办好民生实事。投入扶贫资金15.15亿元，52个易地扶贫搬迁集中安置点开工建设，9.21万人实现精准脱贫，超额完成省定任务。22个重点民生实事项目全面完成。公立医院全部实行药品“零差率”。娄星区在全国首推“农民养老贷”。中心城区活禽交易市场全部搬迁，创建全国文明城市、禁燃与殡改集中整治成效明显。

　　各位代表！

　　本届政府任期的四年，是我市发展史上较为艰难的四年。回首过去，我们既倍感欣慰，更信心满怀。面对“四期叠加”、产业转型和经济下行的多重压力，全市上下风雨同舟、齐心协力，面对困难不退缩、不泄气、不推责，一心干事想法子，甩开膀子抓落实，用“实”的作风和“干”的勇气，顶住了压力，化解了风险，战胜了困难，为“加速转型、奋力赶超”奠定了基础、积蓄了能量，为“变道超车、突围突进”赢得了先机、创造了条件。

　　四年的成绩，可圈可点，来之不易。这些成绩的取得，得益于市委的坚强领导、科学决策，得益于市人大、市政协的有效监督、鼎力支持，得益于全市广大干部群众的齐心协力、顽强拼搏。在此，我代表市人民政府，向全市人民，向各民主党派、人民团体，向驻娄人民解放军和武警、消防官兵、政法干警，向中央、省驻娄单位，向关心支持我市发展的各界人士，表示衷心的感谢和崇高的敬意！

　　各位代表！

　　回顾过去四年，我们也清醒地看到，我市经济社会发展中还存在一些不容忽视、亟待解决的困难和问题:一是经济总量不大，产业结构不优，发展外向度、包容性不强，转型发展处于转折点，面临稳增长和调结构的双重压力。二是民间融资风险，以及由此引发的关联问题，虽然整体可控，但没有根本解决。三是房地产去库存，虽取得了一定成效，但体量大、问题多、情况仍然复杂。四是征地拆迁、控违拆违、“三强”等问题比较突出，发展环境欠优，亟待从制度机制层面加以解决。五是基层基础战斗堡垒作用有所弱化，矛盾和问题得不到及时有效化解，群体性、突发性事件时有发生。六是民生事业、生产安全、生态环境等方面的欠账较多，特别是脱贫攻坚任务十分艰巨。七是城乡基础设施薄弱，城市规划、建设、管理方面的理念与先进地区有差距，标准、品质和水平亟待提升。八是干部作风不实、精神状态不佳等问题，不同程度存在，适应现代经济、招商引资和抓项目建设的能力有待进一步提升，等等。这些问题，是当前制约我市发展的关键性问题，我们绝不能遮掩、绝不能回避、绝不能推诿，必须牢牢坚持问题导向，切实强化忧患意识，以敢于啃硬骨头的精神，一个一个加以攻克。

　　二、未来五年主要目标和任务

　　未来五年，是我国深化供给侧结构性改革、全面建成小康社会的重要时期，是我省实施“创新引领、开放崛起”战略、建设“五个强省”的关键时期，是我市“加速转型、奋力赶超”、建设富饶美丽健康祥和新娄底的攻坚时期。站在新的发展起点，我们要以更加开放的姿态、更加开明的胸怀、更加宽广的视野，立足“一带一路”“一带一部”的战略布局，在全省“一核三极四带多点”的新格局中，找准娄底的发展坐标，积极付诸娄底行动，向“一核”靠拢，为“四带”增色，力争挺进全省市州第二方阵。

　　今后五年政府工作的总体思路是：高举中国特色社会主义伟大旗帜，以邓小平理论、“三个代表”重要思想、科学发展观为指导，深入学习贯彻习近平总书记系列重要讲话精神，认真落实省第十一次党代会和市第五次党代会精神，全面贯彻五大发展理念，以供给侧结构性改革为主线，以“七个三年行动计划”为抓手，坚持转型创新，矢志攻坚克难，坚决打赢转型发展、脱贫攻坚、生态环境治理攻坚战，确保如期全面建成小康社会，努力建设富饶美丽健康祥和的新娄底。

　　未来五年的奋斗目标是：地区生产总值年均增长8.5%以上；固定资产投资年均增长15%以上；一般公共预算收入、地方收入年均增长9%以上；规模以上工业增加值年均增长9%以上；社会消费品零售总额年均增长12%以上；城乡居民人均可支配收入年均增长10%以上；城镇登记失业率控制在4.6%以内，全力争创全国资源型城市转型发展示范城市，打造大长沙拓展区，建设沪昆高铁经济带重要支点城市和山水相融、宜居宜业、众人向往的现代化城市。

　　实现上述目标，我们必须把中央和省委、省政府的战略部署，与我市的具体实际结合起来，按照市第五次党代会的总体要求，切实在四个方面下功夫，致力描绘四幅新画卷。

　　(一)矢志加快转型创新，构建产业新体系。以供给侧结构性改革为主线，坚持创新驱动，加快动能转换，推进产业转型升级。全面振兴工业，着力打造钢铁深加工千亿产业集群，汽车零配件、新能源储能、住宅工业化等百亿产业集群，湖南省汽车配套产业、板材深加工、中部地区不锈钢三大产业基地，农机、煤机、应急产业、精细陶瓷等特色产业集聚区；做大做强有色、建材、化工、轻工等传统产业，大力发展新材料、生物医药、电子信息、生态环保等新兴产业。全面补齐服务业短板，着力培育文化旅游、商贸物流、健康养老、现代金融等四大战略性支柱产业，积极发展电子商务、科技信息、体育会展等新兴服务业，基本建成全国二级物流节点城市和全省旅游热点城市。加快农业现代化进程，以品牌建设为抓手，创新农业发展方式，提高农业比较效益，使农业成为富农民、稳人心的产业。推进三次产业融合，强化“互联网+”思维，鼓励“大众创业、万众创新”，大力发展混合所有制经济，推动新技术、新产业、新业态蓬勃发展，着力提升实体经济整体实力。集中3至5年时间，逐步摆脱资源依赖型发展模式，构建支撑转型发展的现代产业体系。

　　(二)全力推进开放崛起，拓展发展新平台。积极对接“一带一路”“一带一部”战略布局，打造大长沙拓展区、建设沪昆高铁经济带重要支点城市。深入实施融城发展战略，以娄底大道为主轴，推进“娄涟双”“冷新”主副板块纵深发展，加快高铁、高速沿线城镇化建设，基本形成以品质中心城区为龙头，以秀美县城为主体，以特色小镇、美丽乡村为依托的城乡发展新格局。加快万宝新区、东部新区和水府新城建设，拓展城市发展空间。推进“智慧城市”建设，提升城市规划、建设、管理水平，着力打造国内同类型城市中的一流城市。加快实施“娄商返娄”“走出去”战略，推动更宽领域、更大规模、更高层次发展。让娄底走向世界，让世界点赞娄底。

　　(三)深入践行绿色发展，打造生态新优势。全面推进“天蓝水净地绿三年行动计划”和城乡环境综合整治，严守资源消耗上限、环境质量底线和生态保护红线，完成主要污染物减排任务。森林覆盖率达到52%以上，空气质量、地表水质持续提高。建立环境保护负面清单，发展绿色低碳循环经济，推进要素集约节约利用，努力实现生态建设、环境保护与经济发展同步推进，形成人与自然和谐发展的新格局。以对子孙后代负责的态度，加强采煤沉陷区、采矿沉陷区和老工业区的环境修复治理，逐步还老账，绝不欠新账。

　　(四)决战决胜脱贫攻坚，增进人民新福祉。把脱贫攻坚作为第一民生工程，坚决打赢脱贫攻坚战，确保2018年新化、涟源、双峰三个贫困县实现摘帽，2019年所有贫困村和建档立卡贫困人口整体脱贫。持续增加城乡居民收入，不断提高人民生活水平，让全市人民拥有更多的获得感。大力发展社会事业，认真解决群众关心的就业难、看病难、读书难、住房难等问题。健全社会保障体系，推进基本公共服务均等化，让每一个娄底人共享阳光雨露。深入开展“中国梦”和社会主义核心价值观教育，大力弘扬优秀传统文化，提升市民文明素质，全力创建全国文明城市、国家森林城市和金融生态良好城市，着力打造“健康娄底”“平安娄底”“诚信娄底”。让娄底成为全市人民身心向往、情感归依的美好家园。

　　各位代表！

　　当前，我市正处于“加速转型、奋力赶超”的转折点和关键点。加速转型，是没有任何退路的唯一选择；奋力赶超，是全市人民的共同心愿。让我们始终保持“建功必须有我”的担当、“功成不必在我”的境界，肩负起时代赋予的使命，一年接着一年干，一张蓝图干到底，努力把美好蓝图变成生动现实，把政府工作变成人民群众看得见摸得着的实惠。

　　三、2017年工作重点

　　今年，是供给侧结构性改革的深化之年，也是我市推进七个“三年行动计划”的关键之年。必须按照中央、省委和市委经济工作会议精神，一项一项抓好落实。

　　今年的预期目标是：地区生产总值增长8.5%左右，其中一、二、三产业分别增长3.5%、8%、11%以上；固定资产投资增长18%以上，规模工业增长8.5%以上，社会消费品零售总额增长12%以上；一般公共预算收入、地方收入均增长9%以上；城乡居民人均可支配收入分别增长8.5%、10.5%以上；节能降耗完成省定目标任务。着力抓好以下七个方面的工作：

　　(一)着力扩大有效投资

　　强力推进PPP项目。重点围绕国家“7大工程包”，谋划发布一批、加快推进一批，确保市级平台开工建设5个以上，各县市区和娄底经开区、万宝新区分别开工建设3个以上，市直基础设施和公共服务领域开工建设2个以上，年度总投资达到400亿元以上。

　　加快推进重大项目建设。继续开展“项目建设大比拼”活动，完成产业投资640亿元左右，分别完成技改投资300亿元、战略性新兴产业投资60亿元、农业产业化投资60亿元、服务业投资220亿元以上；服务业中，文化旅游健康养老产业完成投资150亿元以上；重大基础项目完成投资380亿元以上，分别完成城市基础设施240亿元、交通70亿元、能源40亿元、信息网络30亿元以上；重大民生项目完成投资280亿元以上。重点推进400个重大项目、60个重特大项目、10个标志性工程项目，确保完成投资800亿元以上。全年谋划重大项目400个以上。

　　以革命性举措优化发展环境。全面开展“征地拆迁控违拆违百日攻坚行动”，严厉打击“三强”行为，精准、高效供地，坚决搬掉阻碍项目落地的绊脚石。加快“智慧娄底”建设，构建“互联网+政务服务”平台，重构议事规则，优化办事流程，提高行政效率，把该管的事管好，把该放的权放掉，让广大投资者安心创业、放手发展。大力营造“四个尊重”的社会风尚，建立引才绿色通道，探索人才柔性流动机制，培养引进科技领军人才、专业技术人才、高技能人才和青年创新创业人才。抓好村支两委换届工作，夯实基层基础，选出想干事能干事的好班子，把矛盾化解在源头，把问题解决在基层。我们要强化“娄底意识”，坚决与娄底发展共荣辱、同进退，不管是党员干部，还是普通群众，都要当项目建设的“促进派”“主攻手”，不要当“反对派”“守门员”，以湘军精神打好项目建设大会战。

　　(二)着力加快转型升级

　　加快打造工业升级版。紧盯“中国制造2025”，推动“娄底制造”向“智能制造”转变。发挥现有产能优势，推动一批技改项目，做大做强精品钢材、薄板深加工、不锈钢产业。加强和长三角、珠三角、大长沙的产业对接与配合，引进一批关联产业和配套项目，着力建设钢铁千亿产业深加工基地，培育汽车零配件、新能源储能、住宅工业化三大百亿产业集群。推进涟源应急产业园建设，促进新化精细陶瓷规模化集群式发展，支持双峰县全国农机工业强县建设，打造全省首家境外农机产业园。抓好煤矿关闭退出工作，年内实现烟花爆竹企业全部退出。

　　全面提升现代服务业。加强城市综合体建设，引导住宿、餐饮、零售等生活服务业连锁化、品牌化、特色化发展，打造一批特色鲜明、影响力强的名街、名店、名品。大力发展全域旅游，加快旅游专线(环线)建设，扩大低空旅游飞行范围，打造全省重要的旅游热点城市，支持新化县、涟源市创建全国全域旅游示范区，年内建设1至2个旅游精品示范区。加快娄底经开区城郊型文化旅游休闲中心、众一健康城、龙山与天门健康养生基地建设。争取湘中国际物流园、诚通物流园竣工投产，支持各县市区加强物流项目建设，启动娄底会展中心建设。重点推进双峰全国电子商务进农村综合示范县、新化省级农村电子商务示范县建设，支持万宝新区国家电子商务示范集聚区、冷水江和涟源全国电子商务强县建设，培育发展一批电商镇、电商村。以社区(村)为单位，建立商贸服务平台，建设一批便民连锁超市。继续开展房地产业“解难题、促发展、保稳定”专项行动，进一步化解竣工扫尾难、交房办证难、库存消化难、税费清缴难等问题，促进房地产业健康持续发展。

　　推进农业供给侧结构性改革。扎实做好全国第三次农业普查，全面摸清“三农”家底。坚守耕地红线，确保粮食总产160万吨以上，蔬菜总产150万吨以上。完成土地确权登记颁证，加快农村土地流转，大力发展农村新型经济组织，以“互联网+”的思维，推动农业供销现代化，新增2家以上销售收入过亿元的龙头企业，确保全市农产品加工企业销售收入增长15%以上。着力培育品牌、突出特色、提升品质，做好农工融合、农旅融合文章，大力发展休闲、观光、体验型农业，促进农业提质增效。

　　打造产业升级版，必须大力振兴实体经济。要以供给侧结构性改革为主线，以提高质量和核心竞争力为中心，立足我市产业基础和资源禀赋，全力推进一批重点产业和特色优势产业发展，增强产品竞争力。大力发展混合所有制经济，毫不动摇鼓励、支持、引导非公有制经济发展，支持民间资本参与公共领域投资和重大项目建设。大力弘扬敢于担当的企业家精神和精益求精的工匠精神，既要大手笔、大气魄，打造百亿、千亿产业集群，又要以质取胜，培育更多的“名优产品”“百年老店”，让娄底大地上大企业顶天立地，小企业铺天盖地。

　　(三)着力建设美丽家园

　　建设品质中心城区、秀美县城。加快万宝新区、高铁新城、娄北新区、水府新城建设，推进城西老工业基地搬迁改造。以重大PPP项目为抓手，推进中心城区“一整二改三化”，基本完成23条新建城市道路及配套设施建设，24条道路白改黑及绿化提质改造。完成中心城区部分区域区划调整，推进冷新融合，加快各县市城区扩容提质步伐。

　　建设特色小镇、美丽乡村。推进高铁、高速、国省干线公路沿线特色小镇建设试点，建设一批金融小镇、产业重镇、旅游强镇、文化名镇和风情小镇，形成可操作可复制的“娄底经验”。以24个省级美丽乡村示范村建设为重点，打造一批“五美”新农村。

　　提升城市规划建设管理水平。以国际国内同类型先进城市为标杆，加快制定和实施“娄底标准”，编制市域城市规划及专项规划，杜绝低水平重复建设，减少维修维护成本。以“智慧娄底”建设为统揽，加快“云上娄底”、大数据中心、电子政务等平台建设，推动城市标准化、网格化、精细化和信息化管理。加大建绿管绿力度，加快“慢行绿道”建设，加强城乡生活垃圾和污水治理。规范物业管理。开展城区治堵专项行动。巩固创园、创卫成果，确保通过国家卫生城市资格复核。

　　(四)着力补齐主要短板

　　补齐县域经济短板。推进产业强县(市)、特色兴县(市)战略，发挥比较优势，破解土地、资金瓶颈，壮大龙头企业，培育支柱产业，发展特色经济，逐步形成“一县一特色产业”的县域经济发展格局。支持各县市区重点打造销售超100亿的主导产业、超50亿的特色产业集群。

　　补齐园区经济短板。加快园区管理体制机制创新，推进简政放权，支持园区开发建设平台集团化改革。积极探索“一区多园”、共建共享新模式，注重引导产业向园区集中，推动园区经济整体提质。深入实施园区倍增提质行动计划和“623”工程，推进“产城融合”，完善科技、金融、创业等服务体系，加快路网、市政等基础配套扩容提质。确保各类园区工业投资、工业税收均增长25%以上。

　　补齐开放型经济短板。树立“海洋意识”，强化“工业思维”，加快主动开放和双向开放，完善服务平台，升级外贸基地，启动娄底保税仓库建设，建成省级出口食品农产品质量安全示范区。紧盯长三角、珠三角、京津冀等重点区域和央企、上市公司，开展精准招商。确保内联引资增长17%以上，实际利用外资增长16%以上。新签约招商引资项目100个以上，其中投资超50亿的项目1个以上，超30亿的2个以上，超10亿的30个以上。引进总部经济企业100家以上。大力发展外向型经济，推动“娄品出境”，确保进出口总额、加工贸易额均增长10%以上。

　　补齐金融短板。坚持积极稳妥、依法依规、分类处置，探索民间融资化解的新办法，引进有实力的公司进行资产处置、破产重组，力争取得实质性突破。加强金融风险防控，加快社会信用体系建设，积极创建金融生态良好城市和省级金融安全区。加快完善现代金融市场体系和服务体系，发展新型金融业态，着力引进各类金融机构来我市设立分支机构和区域性、功能性总部。进一步搭好政银企合作平台，鼓励企业上市，适度做大金融规模。支持万宝新区建设全省金融集聚区，做大做强双峰金融特色小镇，提升金融集聚度。

　　(五)着力实施脱贫攻坚

　　把脱贫攻坚作为“十三五”头等大事和“第一民生”工程来抓，坚决落实“六个精准”“两不愁三保障”要求，高质量推进精准扶贫工作。开展“四跟四走”产业扶贫，提升贫困群众自主发展能力。推进教育扶贫，完成农民技能培训10万人次以上，对贫困学生进行精准资助，实现贫困县农村义务教育阶段学生营养改善全覆盖。强化基础设施与基本公共服务扶贫，启动实施贫困村公共服务设施三年行动计划。加强金融扶贫，提高扶贫小额信贷发放额度。拓展社会扶贫，开展“万企万村”“一家一”助学就业等扶贫活动。加强对五保人员、零就业家庭、因病因学返贫等人员的兜底保障。坚决防止虚假脱贫、口号脱贫、数字脱贫，让贫困群众有实实在在的获得感。

　　(六)着力深化改革创新

　　以更大决心和勇气推进改革。强力推进国企国资等28项省定改革事项，深化投融资等12项自主改革事项。以供给侧结构性改革为主线，推进实施《娄底市资源型城市转型发展系统性改革试点工作方案(2016-2020)》，积极探索“娄底版”改革，力争在产业培育、科技创新、园区管理、城市规划建设管理、农业农村发展、特色小镇建设、征地拆迁和社会治理等方面，取得新的突破。

　　推进以科技创新为核心的全面创新。建立产业技术创新联盟，加强知识产权运用和保护，支持产业共性技术重大项目研发，培育15家以上省级高新技术企业，建设一批国家级、省级众创空间、企业创新平台、技术中心、技术(工程)研究中心、检测中心和计量中心。加大科技投入，改革管理方式，推进“大众创业、万众创新”，实施“质量强市”“娄品工程”，支持涟源高新技术产业园发展，支持冷水江打造国家级农业科技园区。

　　(七)着力增进民生福祉

　　加快发展社会事业。统筹城乡教育一体化建设发展，加大职业教育、民办教育和特殊教育发展力度。继续化解义务教育大班额，切实加快薄弱学校改造和合格学校、公办幼儿园建设。严格落实乡村教师支持计划，适度提高乡村教师待遇，加快教师周转房建设。推进市二人民医院儿科楼、3家妇幼保健院的改造搬迁等项目建设。推进“三医联动”改革，发展中医药事业和产业。坚持计划生育“三个不变”，抓好与全面两孩政策相配套的公共服务体系建设，加大计划生育家庭扶助力度，积极应对人口老龄化。实行“三馆一站”免费开放，开展全民阅读，推进基层综合性文化服务中心“七个一”、市文体中心提质改造和足球城等项目建设。加强文物保护。创作更多富有娄底特色、体现娄底精神的文化艺术作品，打造一批在全省全国有影响力的文化品牌。

　　切实加强社会保障。实施“全民参保登记计划”，加快推进“四险”统一征缴，建立完善基本养老、基本医疗和失业、工伤、生育等保险制度，逐步实现法定人员全覆盖。新增养老床位1000张以上。加快市城区救灾物资储备中心、市殡仪馆搬迁等项目建设。推进农村低保和扶贫标准“两线合一”。扩大住房公积金覆盖范围。大力发展社会福利和慈善事业，健全社会救助体系，关爱空巢老人、留守妇女儿童、特困人员、残疾人员等弱势群体，积极为他们排忧解难。

　　不断创新社会治理。推进司法体制改革，加强法治宣传教育，强化全民法律意识。抓实平安创建，提高防控能力，严管重点领域，整治突出问题。加强积案化解，实现信访与维稳秩序持续好转。落实“四个最严”要求，严厉打击食品药品犯罪，坚决防范源发性、群体性食品药品重大安全事故。全面落实安全生产责任，持续推进煤矿、非煤矿山、道路交通、危险化学品、特种设备、消防、校园、建筑施工等重点行业领域专项整治，防范和遏制重特大事故的发生。做好人防、地震、气象、外事侨务、民族宗教、对台、贸促、档案、保密、地方志、工会、共青团、红十字会、计生协、科协、文联等各项工作。

　　全面推进生态整治。实施“天蓝水净地绿三年行动计划”、湘江保护和治理第二个“三年行动计划”，出台《孙水河保护条例》，实行“河长制”，深入推进资水、涟水、孙水流域沿岸保护治理。加强水府庙水库开发利用与保护，抓好锡矿山、涟钢周边等重点区域环境综合整治示范区建设，大力实施重金属污染耕地应急性修复治理。推进煤矿沉陷区综合治理、矿山修复与治理、农村养殖污染整治。加强绿化造林和森林资源保护，严厉打击毁林、非法采矿、采砂等行为。让娄底蓝天常在、绿水长流、青山常存。

　　加强国防后备力量建设。坚定支持国防和军队改革，务实推动军民融合深度发展，稳步推进后备力量建设调整转型，巩固提升双拥共建成果，积极做好退役军人转业安置工作，着力加强人民防空和民兵基层建设，促进国防建设和经济建设协调发展。

　　2017年，我们要集中精力抓好以下民生实事：一是完成3.2万人易地扶贫搬迁任务，确保11.68万人脱贫，217个贫困村摘帽。二是完成城市棚户区改造26603户，分配公租房13798套，发放租赁补贴16702户。三是全面实施农村建制村全科医生或助理全科医生驻村全覆盖行动计划。四是实现全市偏远山区中小学校WIFI无线网络全覆盖。五是新增城镇就业3.6万人，新增农村劳动力转移就业3.5万人。六是打通吉星路、娄星路、甘桂路等断头路，全面提升中心城区规划建设管理水平。七是全面实现中心城区城乡公交一体化。八是开通长沙-娄底-新化低空旅游观光航线。九是全面实施湘江、资江娄底流域截污和水体治理行动。十是完成娄底大道娄双段、冷新段主体工程，同步实施沿娄底大道自行车慢行系统项目，以及中心城区国际马拉松赛道项目。

　　四、加强政府自身建设

　　打铁还需自身硬。我们必须抓严抓实抓好政府自身建设。

　　坚决破除思想坚冰，努力建设开放政府。以开放的意识、国际的视野、包容的胸怀，搬掉思想保守的“大山”。既善于“引进来”，也敢于“走出去”；既勇于单干打硬仗，也乐于合作促共赢。跳出娄底看娄底，跳出娄底谋划娄底，敢立大目标，敢与先进比，敢跟强者争。只要是有利于娄底发展的事，只要是能造福娄底人民的事，只要是市委、市政府已经决定的事，我们就要大胆地干，撸起袖子加油干，一刻也不耽误地干。

　　切实强化法律思维，努力建设法治政府。尊法守法，坚决做到一切政府行为在法律轨道上运行。强化规则意识，倡导契约精神，让法治成为全社会的基本信仰和价值追求。认真执行市人大及其常委会的决议决定，主动接受人大的法律监督和工作监督、市政协的民主监督，自觉接受社会各界监督。全面推进政务公开，进一步健全重大行政决策的社会稳定风险评估机制，让每一个决策和每一项事业，经得起历史和人民的检验。

　　全面提高工作标准，努力建设效能政府。巩固扩大“两学一做”学习教育和责任心教育成果，加强干部教育培训，提升履职尽责、攻坚克难能力。与国际接轨，向先进看齐，以“攻坚拔寨”的韧劲，制定规划，确定目标，推进工作。立说立行，雷厉风行，求真务实，久久为功，坚决不搞形象工程和政绩工程，让财政的每一分钱都花在刀刃上。发扬工匠精神，一丝不苟、精益求精，把每一项工作、每一个项目，干到最高标准，以一个又一个的单项冠军，赢得全能冠军。

　　始终坚守红线底线，努力建设廉洁政府。坚决落实全面从严治党要求，强化“四种意识”，加强政府决策、议事规则、办事程序等制度建设，树立看得见摸得着的“红线”，划出不可触碰的“底线”，用明规矩打破潜规则。全面履行“一岗双责”党风廉洁建设责任制，抓实作风建设，既坦荡真诚与企业交朋友，又防止眉来眼去和企业做交易，坚决打击权力寻租、灰色交易等腐败行为，着力构建“亲”“清”的政商关系。坚决治理不正之风，从解决发生在群众身边的腐败问题抓起，严厉打击“雁过拔毛”式腐败，做到有案必查、有腐必惩。

　　各位代表！

　　全面建成小康、实现赶超进位，是时代赋予我们的光荣使命，是全市人民的共同愿望。让我们紧密团结在以习近平同志为核心的党中央周围，在省委、省政府和市委的坚强领导下，勇于变革、敢于创新，不忘初心、奋力前行，为建设富饶美丽健康祥和的新娄底而努力奋斗！
